# Personality, abnormal behaviour, and health: An evaluation of the welfare of police horses

**DOI:** 10.1371/journal.pone.0202750

**Published:** 2018-09-05

**Authors:** Ivana Gabriela Schork, Cristiano Schetini de Azevedo, Robert John Young

**Affiliations:** 1 School of Environmental and Life Sciences, Peel Building, University of Salford Manchester, Salford, United Kingdom; 2 Department of Post-Graduate Studies in Zoology, Pontifícia Universidade Católica de Minas Gerais, Belo Horizonte, Brazil; 3 Departamento de Biodiversidade, Evolução e Meio Ambiente, Instituto de Ciências Exatas e Biológicas, Universidade Federal de Ouro Preto, Ouro Preto, Minas Gerais, Brasil; Faculty of Animal Sciences and Food Engineering, University of São Paulo, BRAZIL

## Abstract

An animal’s welfare depends on an individual’s capacity to adapt to the environment in which it lives. This adaptation is directly associated with the quality of the environment and to the possibility of expressing natural behaviours. Horses kept in stables often display a range of abnormal behaviours related to lack of control over their environment, which can lead to behavioural and health problems. An individual’s personality also plays an important role in its susceptibility and resilience to the development of diseases and abnormal behaviour; thus, an evaluation of horses’ personalities could be crucial to selecting individuals best able to cope with different work activities. This study aimed to assess the well-being of police horses maintained in a semi-confinement regime in Brazil by associating their personalities to the occurrence of abnormal behaviours and disease. Using a non-invasive approach, different tests were performed to investigate the horses’ behaviour, personality and welfare. A frustration test and a novel object test were conducted with 46 horses and the individuals’ personalities were assessed using questionnaires and behaviour tests. In addition, we evaluated their physical health through a survey of their veterinary records. The data for horses were evaluated individually. The results demonstrated the occurrence of multiple abnormal behaviours motivated by factors such as diet and lack of social contact. Moreover, the personality tests indicated this is an important component when evaluating welfare, since correlations were found between personality traits and abnormal behaviour expression, and between personality traits and health problems. According to our results, passive, stubborn, and confident horses are better suited to be selected as police horses. The ability to classify horses according to their personalities could help in selecting horses most suitable for patrolling, thereby helping to reduce behavioural problems and increasing animal well-being.

## Introduction

Animal personality can be defined as the variation in an individual’s behaviour that remains stable across time and situations [1]. For animal welfare specifically, understanding how personality is linked to well-being issues could help owners to increase the wellbeing of their animals and reducing economic losses [2]. Abnormal behaviours, for example, are associated with low levels of welfare and are known to compromise an individual’s physical condition [3–5]. Hence, if a certain personality trait is associated with the occurrence of abnormal behaviours, consequently, these individuals will have a higher risk of presenting health problems in the future. Therefore, investigating the relation between abnormal behaviours and the assessment of an individual’s personality could be used as a tool to predict the occurrence of illness [6]. Depending on its purpose, animals could even be select based on the best personality profiles, avoiding problematic outcomes; a common practice for humans [7] and already being used for dogs [8].

An individual will develop its personality, partially; as a consequence of the life it lives [9]. Life situations considered “not normal” (i.e. social animals living in isolation) will produce inappropriate behaviours, meaning that biological conditions can directly affect an individual’s personality and produce abnormal behaviours [10]. For example, research has found that horses considered more reactive (instead of normal or calm) by their handlers tended to display twice as much abnormal behaviour than calm horses [11].

Horse stables differ considerably from the natural environments in which horses evolved and limit their control over different situations [3, 12]. Horses still maintain many of their ancestral behaviours and the limitation of the expression of these behaviours is associated with the development of various animal welfare problems [3, 13–14].

Studies with horses have shown these animals have a complex personality structure with dominant, anxious, excitable, sociable, protector and inquisitive were some of the profiles recorded [15–16]. Moreover, the personality of the horse was found to be important for equestrians, veterinarians and trainers, and should be taken into account during breeding programs depending on the type of activities the horses would be designated to undertake [15–19]. Working stressors are known to elicit the exhibition of abnormal behaviours in horses [20], but no studies relating personality traits, abnormal behaviours and illness in horses have been conducted until now.

The most common and recurrent abnormal behaviours in horses are divided in two categories: oral and locomotors abnormal repetitive behaviours. Among them, crib-biting and weaving prevail over others [14, 21]. There are numerous problems associated with the presence of abnormal repetitive behaviours in horses, such as: colic, weight loss, pain, chronic diseases, self-mutilation, agoraphobia, claustrophobia and maternal rejection [3, 13–14, 22–23].

Studies show that the personality of the animals can be used to predict future health problems [24] as well as their productivity [25]. More neophobic, less-exploratory rats are more likely to develop diabetes and hypertension than more exploratory and less neophobic rats [26–27]. More dominant, aggressive, and impatient humans are prone to develop heart diseases [28]. Happier dogs show a lower incidence of gastric dilatation-volvulus [29]. A set of personality tests has been developed for dogs aiming to select individuals for different tasks [8]. The same could be done for horses [20].

Animal personality can be assessed by behavioural observations or by human observer trait ratings [1, 30–32]. Behavioural observations include novel object test, the open field test, the novel environment test and the emergence test [1, 31–32]. Human observer trait rating uses questionnaires containing pre-defined characteristics to evaluate an animal and usually are conducted with people familiar with the subjects (i.e. caretakers) [1, 31–33].

Tests can also be applied to evaluate the behaviour of animals when experiencing frustration, which could be linked to the animal’s personality. A sham-feeding test is used to evaluate satiety and is represented by the presence of mastication and saliva without food in the oral cavity) [34–35]. The presence of sham feeding, in some cases, is related to frustration due to the absence of food and can lead to abnormal behaviours [36–38]. In horses, food deprivation is linked to the increase of acidification in the digestive system, which causes abnormal behaviours such as crib-biting [13]. Therefore, a sham-feeding test could be used to evaluate the potential expression of abnormal behaviours in horses.

Currently in the state of Minas Gerais Brazil, horses are used by the police as the main source of labour for urban patrolling and previous studies with these animals have shown high incidence of abnormal behaviour, colic and other diseases [39–41]

Further research conducted with police horses also demonstrated that this specific type of work could significantly affect the physical and behavioural responses of these animals [42–43].

Considering the welfare of police horses, this study aimed to evaluate the personality and wellbeing of Brazilian police horses using behavioural and physical health variables. To investigate if the horses’ personalities predicted the expression of abnormal behaviours and the incidence of disease.

## Methodology

### Study site

This research was developed at the police horse headquarters, Regimento de Cavalaria Alferes Tiradentes–PMMG (RCAT—19°55'18.35" S 43°57'48.07" W) located in the city of Belo Horizonte, state of Minas Gerais, Brazil.

### Ethical disclaimer

All the behavioural tests with the horses were considered non-invasive and followed the internal regulations and horse management practices of the police. The tests were accompanied by either a veterinarian or a veterinary nurse at all times. At the time this study was conducted, no ethical approval was required for non-invasive research by the University’s Ethics Panel (PUC Minas) or the Police (PMMG) (S1 Fig).

### Horses, housing and maintenance

For this study, 46 adult horses, 20 neutered males and 26 females used for urban patrolling, with regular working shifts, were tested. The horses belonged to the Brazilian Sport Horse breed, with a mean age of 10.37 (± 0.67 SD) years and were being used for urban patrolling for at least one year prior to the start of the study. The normal work load for the horses consisted of an 8-hour shift every other day: in cycles of 45 minutes patrolling followed by 15 minutes of rest. When not patrolling the horses were maintained in stalls and did not perform other activities.

The horses were individually housed in 2 x 2.5m masonry stalls with wooden doors and cement floors (no shavings/bedding). The animals were fed six times a day, alternating 2kg of hay and 2kg of a concentrate mix of grains, and had access to water *ad libitum*. The horses had weekly veterinary health check-ups.

### Data collection

Data collection was divided in four different phases. All data were collected on days when the horses were not on duty.

#### Sham-feeding test

Based on previous research with pigs [37–38] that directly associated food restriction (physical) with the expression of stereotyped behaviours, we developed a Sham-feeding test with the horses, where a qualitative rather than a quantitative restriction of a food reward was made. This test was applied to evaluate the rate of abnormal behaviours expressed by the horses.

The sham-feeding test was divided in to three six-minute sessions where the horses were given or not given a food treat (brown sugar cubes). The horses were tested in pairs and individually and the behavioural responses were recorded through a portable video camera (Sony DCR-HC52) mounted on a tripod.

All animals were subjected to the three sessions, on different days, with at least two weeks’ interval between each session. The test procedures were:

**Session one:** a pair of neighbouring horses was tested simultaneously, one being the test subject and the other one the control subject. For the first minute, at every 15 seconds interval, both horses were given the food treat. From minute one to minute three, the control horse continued to receive the treat every 15 seconds while the test horse was presented with an empty hand. After the third minute, the researcher performing the test left the test area and the horses continued to be filmed for three more minutes (in total a six minute session). This procedure would provoke frustration in the horse that did not receive the food treat, increasing the chances of the expression of abnormal behaviours [44–45].

**Session two:** the sham test was repeated, but instead of a pair of horses, one of the horses was tested alone. The neighbour horse previously used for control was removed from its stall until the test was finalized. The tested horse was again presented with a treat every 15 seconds for the first minute, then the next two minutes it was presented only with an empty hand and after the third minute, the researcher left the area and the behaviour of the horse was recorded until completing six minutes. This procedure would provoke frustration in the test horse, increasing the chances of the expression of abnormal behaviours.

**Session three:** the test conducted in session one was repeated with the same pair of horses, but the roles of the individuals were reversed.

After the completion of all tests (N = 138, for 46 horses, 18 minutes per test per horse), the video recordings of the sessions were analysed and the behaviour of the horses was registered using focal sampling with continuous recording of behaviour [46].

#### Personality questionnaires

To access the individuals' personalities, a questionnaire was constructed using 18 personality traits adapted from previous studies with horses [47–48] (Table 1). The animals were ranked by four evaluators (three veterinarians and the horse’s rider), for each personality trait, on a scale from 0 to 7, where zero meant no expression of the trait and seven, the total expression of the trait [49]. All evaluators had continuous contact with the horse for at least one year. The questionnaires were answered individually and the evaluators were asked not to comment on their results to other evaluators.

**Table 1:** Personality traits used in the questionnaires applied to police cavalry officers and veterinarians to evaluate horses’ personalities.

Personality traits	Behavioural description
**Active**	Constantly moving, is not observed standing still for long.
**Aggressive**	Displays signs of aggression towards humans and/or horses, can cause harm to another individual.
**Confident**	Behaves in an assured manner, does not easily hesitate.
**Curious**	Explores new situations without hesitation.
**Equable**	Easy to handle, remains calm around other horses and/or people, behaves gently.
**Insecure**	Hesitant when alone, is reassured by the presence of others, seems more confident among others.
**Irritable**	Does not tolerate disturbances, responds negatively if provoked.
**Opportunistic**	Takes advantage of situations as they arise.
**Playful**	Initiates and/or takes part in play when requested.
**Passive**	Behaves in a relaxed manner, not easily disturbed, is slower than other horses when handled.
**Sociable**	Seeks company of others, behaves in a positive and appropriate way around other horses and/or people.
**Stubborn**	Does not cooperate easily, takes time when performing tasks, does not easily give in.
**Intelligent**	Learns new things easily, is faster than others in responding to mental tasks.
**Solitary**	Prefers to be alone when a group interaction is possible.
**Hardworking**	Responds promptly and appropriately when given tasks, remains focused on what is required to do.
**Fearful**	Is startled easily, does not react well to new situations, tries to escape from disturbances.
**Reliable**	Can be trusted to perform tasks, is consider a safe horse to be around.
**Cooperative**	Horse is easily conducted, does not demonstrate resistance.

#### Novel object test

To evaluate the behavioural responses of the horses to an unknown stimulus, and to evaluate aspects of horse’s personality, a novel object test was conducted. The tests were conducted at the horse’s own stall. A metal basket was adapted and attached to the wooden door of the horse’s stall and novel objects were placed inside the basket. To avoid any bias, the horses were habituated to the basket before the introduction of the new items. Each animal was allowed to investigate the basket for 15 minutes before the test commenced. After this period of time, the attention of the horse was redirected to the rear end of the stall, were it was given a food reward by one of the veterinarians (from the back corridor), and in the meantime the novel object was placed in the basket. Once the object was in place, the veterinarian left and as soon as the horse turned and started to face the front of the stall, the test began.

The animals were observed for a total time of 10 minutes. Observations were made using focal sampling with instantaneous recordings of the behaviour every 10 seconds [46] and data collection was based on an ethogram for horse behaviour developed from two previous studies [50–51] (Table 2).

**Table 2:** Ethogram for horses used in a novel object test.

Behaviour*	Description
**Standing active**	Animal is standing still, but shows activity, such as hoof or head movements.
**Standing resting**	Animal is standing but shows rest positions. E.g. One of the hind legs flexed, ears pointing down and to the side or, lower lip relaxed (dropping), eyes closed or partially closed.
**Alert**	Animal is standing still with the, head standing high. Ears are erect and pointing forward. The nostrils may or may not be dilated.
**Locomotion**	Animal moves using its limbs. E.g. straight line or in circles.
**Alarm movements**	Animal displays an alert posture with members stretched and rigid, open nostrils, ears pointing in the direction of the stimulus. Can accompany abrupt movements of escape attempt, like small jumps and/or alarm vocalizations (Winch).
**Interacting with object**	Any interaction performed by the animal with the object presented in the test E.g.: sniff, lick, bite, etc.
**Observing object**	Animal carefully observes the object with ears up and pointing to the direction of the object
**Interacting with the basket**	Any interaction by the animal related to the basket but not with the object E.g.: sniff, lick, bite, etc.
***Flehmen***	Animal stands still, raises the neck and tilts the head back with the ears upside down, while everting the upper lip, making evident of the incisor teeth and the upper gum. Behaviour associated with olfactory exploration.
**Exploration**	Olfactory and tactile exploration of the environment. E.g. Horse searches for food on the ground or in different locations of the stall, other than the feeder. Usually touches the environment using the upper and lower lip.
**Abnormal behaviour**	This category includes any behaviour that derives from a normal pattern for the animal and repetitive abnormal behaviours. E.g.: air swallowing, weaving, pacing, lip smacking, tongue playing, lip-twisting, crib-biting, stomping, pawing, head-shaking.
**Social interaction**	Interactions with other horses through physical contact. Eg: touch of snouts, mutual grooming, etc.
**Agonistic behaviour**	Aggressive social interaction or aggressive displays towards another horse and/or person. E.g. bites.
**Vocalization**	Characteristic sounds emitted by the animal with short or long duration.
**Eat**	Animal ingests food portions, terminated with the end of mastication and swallowing. It also includes activities such as licking salt blocks.
**Drink**	Animal sips and swallow water.
**Maintenance**	Urination, defecation and grooming related behaviours, such as scratching, licking the fur, etc.
**Other**	Any other behavior displayed by the animal that does not fit in any other categories.

#### Health assessment–veterinary records

The last step in evaluating the welfare of these horses, was a survey of their veterinary records and any pathology that could be associated with behavioural problems (abnormal behaviours) [41] was recorded.

### Statistical analyses

#### Sham-feeding test

The normality of the data was tested through an Anderson-Darling test. The data were classified as non-parametric and Generalized Linear Mixed Models (GLMMs) were built to verify if abnormal behaviours (dependent variables) were affected by the three phases of the sham test or by the individual horses (explanatory variables). All the tests were conducted in Minitab® 18.1 [52] with 95% confidence interval (S1 Table).

#### Horses personaltity

To evaluate the composition of the horses’ personalities, different analyses were conducted combining the data from the personality’s questionnaires, the results of the novel object test and the results of each phase of the sham-feeding test.

According to other studies, the great variation in the assessment of an individual’s personality through a questionnaire relies on different evaluators [49]. Hence, Kendall’s coefficient of concordance (W) [53] was calculated to verify raters’ agreement and reliability. This coefficient varies from 0 to 1 and a score closer to one means a higher association [53]. The coefficient was calculated for each horse and each one of the adjectives among the four raters. When the Kendall’s coefficient of concordance for a horse trait’s was non-significant (p>0.05), the data were analysed using a Spearman’s rank correlation to identify if any of two evaluators had agreed on their scores. All the adjectives that failed to find any significant correlation among at least any two raters were considered unreliable and discarded from further analysis [54]. After the removal of the non-significant personality traits, the Kendall’s coefficient was recalculated and the final (W) value was considered as the concordance rate among the raters.

Following this, a mean of the reliable adjectives was calculated for each animal using the scores marked by the raters. The obtained values were then analysed in a Principal Component Analysis (PCA) with a Varimax rotation. The extracted components were determined using the eigenvalue criteria (the value must be greater than one) and the percentage of contribution of each one of the adjectives to the components was verified. Additionally, during the PCA, the scores of the components for each horse were calculated using the adjectives’ loadings.

To investigate if the personality traits had an association with the recorded diseases (colic and lameness), Generalized Linear Models (GLMs) were built to verify if the occurrence of diseases (dependent variables) were affected by the horses’ personalities (explanatory variables). Also, Spearman’s rank correlations were calculated among the results to verify if any personality trait varied with the occurrence of the diseases. These analyses were run using both the mean values obtained from the questionnaires and the residual loadings from the PCA with a quadratic transformation (trait measures).

Furthermore, the mean frequency of the observed behaviours in the novel object test and during the three phases of sham-feeding test were compared against the adjective loadings for each component obtained in the PCA analysis for each horse. Both PCA analysis and the Spearman’s rank correlation tests were carried out in SPSS® 20.0 [55] with 95% confidence interval. The GLM was carried out using Minitab® 18.1 [52] with 95% confidence interval (S2 Table).

#### Veterinary records

Data regarding colic and lameness cases whose cause was unknown by the veterinary staff were selected from the horses’ veterinary records—the records covered any occurrence in the period between the horse arrival to the unit up to six months prior to the study. An individual’s health rate was created using the number of colic cases and number of lameness cases divided by the total number of years each horse was in the police. Additionally, a mean rate was calculated for all animals for both pathologies. Spearman’s rank correlations were calculated between each individual’s health rate (colic and lameness) and the mean frequency of abnormal behaviours observed for each horse during the three phases of the sham-feeding tests.

## Results

### Sham-feeding tests

During the three phases of the sham-feeding test, it was possible to identify 10 distinctive abnormal behaviours expressed by the horses: Head-shaking, Lip-smacking, Tongue playing, Licking, Crib-biting, Kicking, Pawing, Stomp, Lip-twisting and Weaving. These abnormal behaviours accounted for 16.75% of the behaviours observed during the sham-feeding test, which corresponded to >15% of the total test time. However, the expression of abnormal behaviours was not affected by any phase of the sham-feeding test (P > 0.05), but it was affected by the individual variation (Table 3).

**Table 3:** Generalized linear mixed models results showing that the abnormal behaviours exhibited by police horses during the sham feeding experiments were influenced by individual variation.

Abnormal Behaviour	DF	Deviance	Z	*p*-value
Head-shaking	45	0.00078	3.87	<0.001
Lip-smacking	45	0.00003	4.17	<0.001
Tongue playing	45	0.00015	4.41	<0.001
Licking	45	0.00036	3.05	<0.001
Crib-biting	45	0.00001	1.64	<0.05
Kicking	45	0.00028	0.42	<0.001
Pawing	45	0.00026	4.74	<0.001
Stomp	45	0.00003	2.54	<0.01
Lip twisting	45	0.00041	4.31	<0.001
Weaving	45	0.00020	3.40	<0.001

DF = degrees of freedom.

The horse that exhibited most abnormal behaviours during the tests expressed these behaviours during 14:14:04 minutes (59.31% ± 8.98 SD) of the total test time (18 minutes). The most expressed abnormal behaviour by this individual was lip-twisting (26.59% ± 5.79 SD). When evaluating individual variation during the three phases of the sham-feeding test, ‘head-shaking’, ‘crib-biting’ and ‘licking’ were the behaviours that had the greatest variation between horses, with horses number 28, 15 and 12 showing significant differences (p<0.05). The behaviour with the least number of horses varying between the three phases of the test was ‘lip-smacking’, with only one horse being significantly different from the others (p<0.05).

### Personality tests

Twelve of the 18 personality traits showed positive concordances among the raters (W = 0.394, N = 46, p < 0.001). Six personality traits were excluded from further analysis because they did not reach concordance with at least two raters (confident, opportunistic, passive, sociable, fearful and solitary).

**Fig 1:**
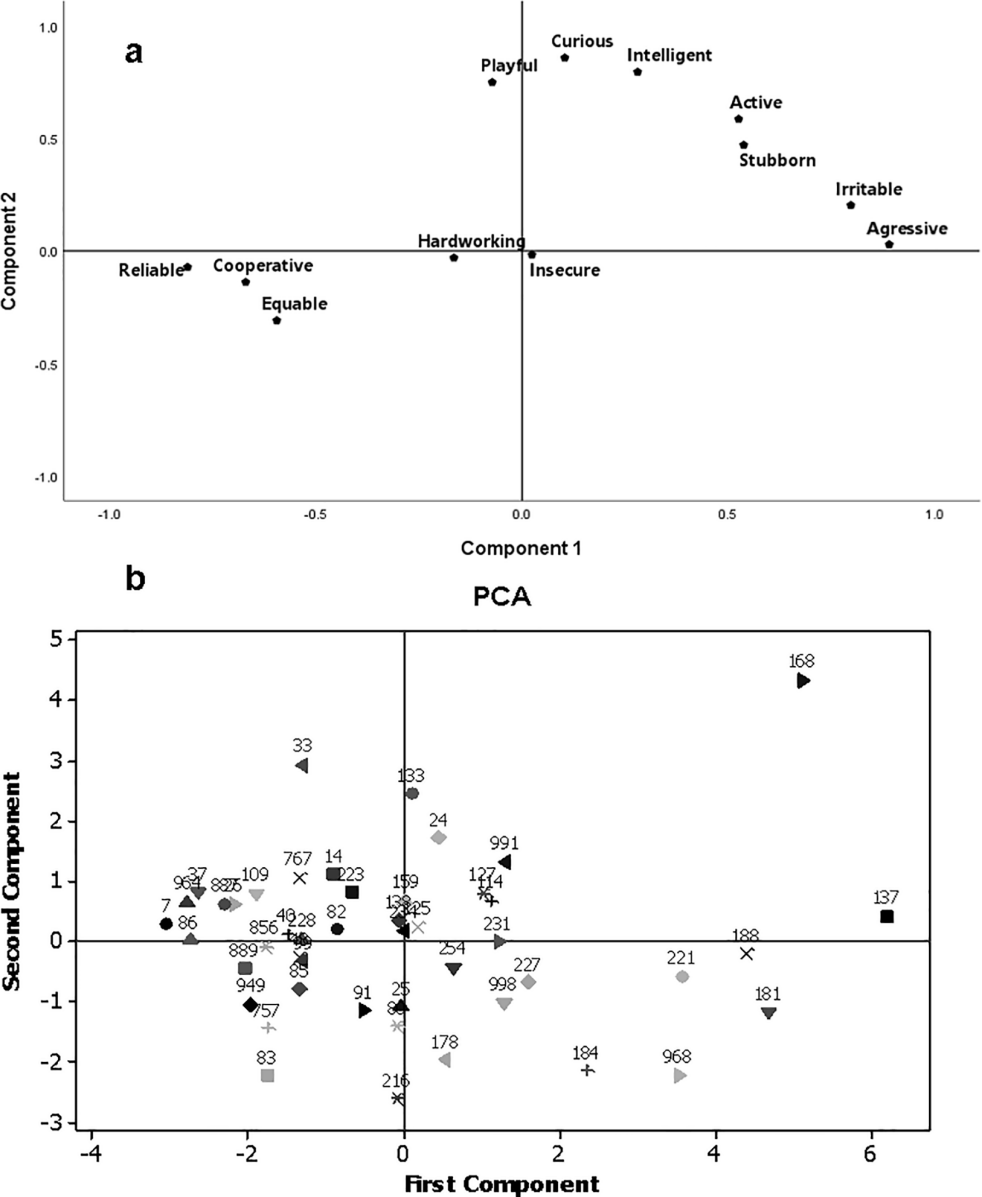
A. **Personality traits of police horses in relation to the first two components of the Principal Component Analysis with Varimax rotation**. The first component was related to the traits aggressive, reliable, irritable, cooperative, equable and stubborn, and the second component was related to curious, intelligent, playful and active. B.**Spatial distribution of the loadings presented by the 46 horses in relation to the first two components of the Principal Component Analysis with Varimax rotation according to their personality traits**.

Four components were extracted from the Principal Component Analysis with the remaining horses’ personality traits. These four components explained 77.52% of the total variance and presented eigenvalues greater than one. The traits aggressive (+), confident (-), irritable (+), cooperative (-), equable (-) and stubborn (+) presented their highest loadings in the first component and were associated with the perception of the raters about the horses behaviour during husbandry/patrolling (Fig 1A; Table 4).

**Table 4:** Loading values of personality traits extracted by the Principal Component Analysis with Varimax rotation using the mean scores given to police horses by their rider and veterinarians.

	PCA Components			
Personality Traits	1^π^	2^¥^	3^β^	4^Δ^
**Aggressive**	**0.890**	0.028	0.055	-0.100
**Reliable**	**-0.811**	-0.073	0.391	0.015
**Irritable**	**0.797**	0.202	0.007	0.227
**Cooperative**	**-0.670**	-0.139	0.478	0.265
**Equable**	**-0.595**	-0.310	0.288	0.468
**Stubborn**	**0.537**	0.471	-0.373	0.272
**Curious**	0.104	**0.858**	-0.058	0.015
**Intelligent**	0.280	**0.795**	0.160	-0.194
**Playful**	-0.073	**0.750**	-0.373	0.075
**Active**	0.525	**0.586**	0.361	-0.184
**Hardworking**	-0.165	-0.032	**0.868**	-0.095
**Insecure**	0.024	-0.018	-0.116	**0.940**

Bold values represent the highest load for each adjective among the four components.

π: Component associated with the perception of the raters about the horses’ behaviour.

¥: Component associated with the horses usual responses to their environment.

β: Component related to the horses obedience to the police commands.

Δ: Component related to the horses’ insecurity in its behavioural responses.

The traits curious (+), intelligent (+), playful (+) and active (+) presented their highest loadings in the second component and were associated with the horses usual responses to their environment. Finally, the traits hardworking (+) and insecure (+) presented their highest loadings in the third and fourth components, respectively. The third component related to the horses’ obedience to the police commands and the fourth component related to the horses’ insecurity in its behavioural responses (Fig 1B; Table 4).

Eight horses presented different personalities when compared to others (individuals 33, 168, 137, 188, 181, 221, 968 and 184); they did not group with the others in the PCA analysis (Fig 1B). In addition, these were horses that presented the lowest number of veterinary problems and presented the lowest number of abnormal behaviours.

Some abnormal behaviours were positively associated with specific personality traits. For the sham-feeding test, the abnormal behaviours licking and lip-twisting had a medium-strength negative correlation with the personality traits hardworking and reliable, respectively (licking: r_s_ = - 0.379, N = 46, p < 0.01; lip twisting: r_s_ = - 0.389, N = 46, p < 0.01). The abnormal behaviours lip-twisting and pawing had a medium-strength positive correlation with the personality traits equable and cooperative, respectively (lip twisting: r_s_ = 0.432, N = 46, p < 0.01; pawing: r_s_ = 0.319, N = 46, p = 0.03).

For the novel object test, the sum of abnormal behaviours and a few abnormal behaviours individually (oral abnormal behaviours: lip-smacking, tongue playing, lip-twisting, licking and crib-biting; locomotors abnormal behaviours: head-shaking, pawing, kicking, stomp and weaving) showed correlations with different personality traits. The locomotors behaviours were weakly positively correlated to curious (r_s_ = 0.285, N = 46 p = 0.05) and showed a medium positive correlation with intelligent (r_s_ = 0.400, N = 46, p = 0.002). The oral abnormal behaviours were weakly positively correlated to the cooperative personality trait (r_s_ = 0.285, N = 46, p = 0.05) and the sum of abnormal behaviours showed a medium-strength positive correlation with intelligent (r_s_ = 0.439, N = 46, p = 0.006).

Individually, the abnormal behaviours licking, lip-smacking and stomp showed a medium negative correlation with the trait aggressive (r_s_ = -0.332, N = 46, p = 0.024) and insecure (r_s_ = -0.324, N = 46, p = 0.028) and a weak negative correlation with irritable (r_s_ = -0.035, N = 46, p = 0.023). The behaviours kicking and crib-biting showed a medium-strength positive correlation with curious (r_s_ = 0.304, N = 46, p = 0.040) and playful (rs = 0.339, N = 46, p = 0.021), respectively. Head-shaking was positively correlated (medium-strength) to playful (r_s_ = 0.349, N = 46, p = 0.017), stubborn (r_s_ = 0.309, N = 46, p = 0.037), intelligent (r_s_ = 0.377, N = 46, p = 0.010) and hardworking (r_s_ = 0.309, N = 46, p = 0.037), this was the behaviour that had the most significant correlations with personality traits.

### Novel object test

The most frequent exhibited behaviours during the novel object tests were: interacting with the object (33.60% ± 0.036 SD) and alert (19.84% ± 0.019 SD), followed by standing active (14.26% ± 0.027 SD) and exploration (10.08% ± 0.013 SD). Abnormal behaviours totalled 5.3% (± 0.012 SD) of the recorded behaviours.

Latency to react to the presence of the novel object was 59.1s (± 19.2 SD) and to interact with the novel object was 120s (±15.1 SD). No correlations were found between the type of novel object presented and the latencies to react and to interact with the objects (p > 0.05).

Nine behaviours expressed during the novel object test were correlated to eight personality traits. The personality traits active and playful correlated positively with three behaviours, irritable correlated positively and negatively with two behaviours, and curious, insecure and equable correlated positively or negatively to only one behaviour (Table 5).

**Table 5:** Significant correlations between personality traits obtained from the Principal Component Analysis and the behaviours expressed by police horses during a novel object test.

Traits	Behaviours								
	SA	AM	V	IO	EX	AB	AGO	E	O
**Active**	rs = 0.34 p = 0.03		rs = -0.31 p = 0.04			rs = 0.38 p = 0.01			
**Curious**		rs = -0,31 p = 0.05							
**Equable**								rs = 0.36 p = 0.02	
**Insecure**				rs = -0.31 p = 0.05					
**Irritable**						rs = -0.32 p = 0.04			rs = 0.32 p = 0.04
**Playful**					rs = -0.36 p = 0.03	rs = -0.41 p<0.01	rs = 0.33 p = 0.03		

SA: standing active; AM: alarm movements; V: vocalization; IO: interacting with test object; EX: exploration; AB: abnormal behaviours; AGO: agonistic behaviours; E: eat; O: other behaviours.

### Veterinary records

Only 12 horses out of the 46 individuals did not have any veterinary records of disease (i.e., had never required treatment), 156 veterinary records were analysed and 131 colic cases and 25 lameness cases were found. The maximum number of cases of colic for a single horse was 30; the maximum number of cases of lameness for a single animal was nine. The mean rate of colic per animal was 2.84 (+0.437) and the population rate was 0.29 cases. For lameness occurrences, the mean rate was 0.54 (+0.126) per animal and the population rate was 0.03 cases per animal per year.

Negative weak correlations were found between the abnormal behaviours and the rate of lameness of the horses, both for all recorded abnormal behaviours (r_s_ = -0.189, N = 46, p = 0.027) and for grouped locomotor abnormal behaviours (r_s_ = -0.17, N = 46, p = 0.046), but no correlations were found between the occurrence of lameness and the expression of each behaviour individually.

The occurrence of colic in the horses showed the opposite, with no correlation with the grouped abnormal behaviours, but negative weak correlations with licking (r_s_ = -0.248, N = 46, p = 0.004) and crib-biting (r_s_ = -0.202, N = 46, p = 0.018), and positive weak correlations with lip twisting (r_s_ = 0.186, N = 46, p = 0.030) and tongue playing (r_s_ = 0.199, N = 46, p = 0.020).

Personality traits explained the occurrence of lameness in the horses, when analysing both the questionnaires and the PCA loadings. Curious, playful and intelligent horses presented more lameness than the other horses, both in the questionnaire analysis and in the behavioural analysis. The personality traits active, cooperative, irritable, reliable and equable influenced the occurrence of lameness in the horses when only the ratings from the questionnaires were taken into account, and the personality trait aggressive influenced the occurrence of lameness in the horses when only the behavioural measures were taken into account (Table 6).

**Table 6:** Generalized linear models results showing that the horses’ lameness cases were influenced by the horses’ personality traits. The results are shown for the questionnaire ratings (veterinarians and riders) and for the behavioural measures (PCA loadings with quadratic transformation).

Personality trait	Questionnaire	Behaviour
**Active**	F = 73.81; p < 0.001	-
**Curious**	F = 66.77; p < 0.001	F = 8.71; p = 0.005
**Equable**	F = 8.18; p = 0.007	-
**Irritable**	F = 5.73; p = 0.02	-
**Playful**	F = 40.03; p < 0.001	F = 6.15; p = 0.02
**Intelligent**	F = 6.70; p = 0.01	F = 4.43; p = 0.04
**Reliable**	F = 16.36; p < 0.001	-
**Cooperative**	F = 17.04; p < 0.001	-
**Aggressive**	-	F = 5.57; p = 0.02

N = 46 in all cases; DF = 45 in all cases

## Discussion

The horses exhibited abnormal behaviours in rates varying from 5 to 15% during the different tests and many of these behaviours were associated with personality traits. The most curious, cooperative and intelligent horses exhibited more abnormal behaviours than the more passive and stubborn ones. Aggressive, insecure, irritable and hardworking horses also presented more abnormal behaviours than the horses with confident and reliable traits. In general, more intelligent, playful and curious horses presented more lameness than horses with other personality traits.

During the sham-feeding tests, the expression of abnormal behaviour was always associated with the presence of food, with horses increasing the expression of abnormal behaviours, between the three phases of the test. Some researchers performed restriction tests and found an increase in crib-biting in horses after the withdrawal of the food reward offered to them [11, 56–57]. Similarly, in another study, an increase in the expression of weaving was observed in horses that received more food items during the day than the controls [58]. The occurrence of abnormal behaviours due to food restriction has been observed in other species, such as pigs [37–38], piglets [59] and cows [60]. For horses, it is suggested that the development of abnormal behaviours responses to food are linked to anxiety and anticipation [61].

Although the incidence of abnormal behaviours was high during the sham feeding tests, more than 16% in only 6 minutes, it is known that these behaviours were not developed in response to the sessions, only stimulated. This means that the conditions in which the horses live are a contributing factor for the development of such behaviours [11, 14, 62–63].

Police horses are trained to cope with conflict (e.g., crowds, loud noises, etc.). In the horses studied, this training lasts one year and it is conducted with foals at the age of two. In a natural environment, this is usually the age where the foals are developing their social bonds as they leave their maternal groups [64–65]. Considering these extreme aversive stimuli, the training schedules and the social isolation during training, behavioural problems in these horses are induced; it has been suggested that the suppression of emotions by horses while training contributes to a decreased welfare [20]. These animals are evolutionarily programmed to escape situations like these, not to confront them [65]. Hence, this type of routine could induce the occurrence of abnormal behaviours, especially in foals [20]. The fact that the most curious, cooperative and intelligent horses exhibited more abnormal behaviours reinforces the idea that the demanded obedience during training and daily patrols could cause welfare problems to these individuals. Particularly, in our subjects, along with the non-ideal environment, personality appears to be a key factor determining how the animals’ responded to their environment.

For the studied horses, the first Principal Component loaded traits were more related to extrinsic factors, how the rider perceived the horses’ behaviours, while the second component loaded traits more related to intrinsic factors or how the animals’ naturally behave in the environment (not the perception of the riders and/or during duty). In the first component, the horses were more aggressive, irritable, confident, cooperative, equable and stubborn, and in the second component, the horses were more curious, intelligent, playful, and active. Similar results were found in other studies [47, 66–67]. These results point to a variation in the composition of the horses’ personalities, affected by both extrinsic and intrinsic factors. This variation can be due to a high exposure to various stimuli throughout the horses’ lives, such as number of riders, number of owners, type of work and husbandry [68–71]. Age, breed and genetic factors may also be responsible for differences in horses’ personalities [15–16,70].

In our study, the expression of abnormal behaviours was also correlated with personality traits. In general, intelligent, cooperative, curious, equable and playful horses were more prone to express oral (crib-biting and lip-twisting) and locomotor (head-shaking, kicking and pawing) abnormal behaviours than others.

The reason why intelligent and curious horses tend to develop more abnormal behaviours is yet not clear, but it was suggested that these horses could learn from other individuals that display abnormal behaviours [72], similar occurrences have been found in other species [73–74]. In the same manner, hardworking and cooperative horses are normally more obedient, and these characteristics are linked to the commands received during training, husbandry and work making them more responsive to the environment and more susceptible to develop abnormal behaviours [11]. In fact, there were many anecdotes by the riders in our study, when completing the questionnaires, about the expression of abnormal behaviours by the horses during training sessions.

Lastly, aggressive and irritable horses are known to develop repetitive abnormal behaviours because they are not able to deal with the frustration of a restricted environment, such as stabling [72, 75–76]. In the present study, aggressive and irritable horses showed less expression of licking, lip-smacking and stomp. Maybe these horses did not possess higher levels of anxiety that could culminate in an abnormal response, thereby dealing better with stress [72].

The behaviours expressed by the horses to the novel objects indicated that the objects functioned as environmental enrichment items. They were alert when novel objects were available and interacted with them, which could be interpreted as curiosity towards the objects or as fear. Increased vigilance behaviours during novel object tests were observed in other studies [51, 77–78]. However, due to the short latency time taken to interact with the objects and to the great amount of time spent interacting with the novel stimulus, we interpret their response as curiosity.

In our study, active, equable and curious horses explored more their stalls during the test and ate more, were less alarmed in the presence of novel objects, while horses that were more insecure interacted less with the objects. Corroborating our results, a study showed more insecure horses avoided novel objects [79]. Contrary to this, if a horse does not consider the novel object as a threat, the chance of interaction with the object or of expressing calm behaviours, such as feeding, increases [80]. Active horses showed the propensity to express more abnormal behaviours, whereas irritable and playful horses showed the propensity to express less abnormal behaviours. Usually, active horses maintained in small, barren stalls can redirect their motivation to express abnormal behaviours [81].

The associations found in our novel object tests did not have a strong correlation with the personality assessment. This may be due to the fact that an individual’s behaviour is affected by more than one personality trait and varies greatly between individuals [66–67]. To test this fully would require a much larger sample size.

Finally, when comparing the horses’ health and clinical records with their individual expression of abnormal behaviour, we were able to identify that the expression of locomotor and abnormal behaviours (grouped) presented a weak negative correlation with the incidence of lameness. Colic cases were weakly negatively correlated with the expression of licking and crib-biting, but positively correlated with lip twisting and tongue playing. Previous studies have shown that the occurrence of colic in horses is directly associated with crib-biting and air swallowing [22–23]. Usually, the horses expressed lip twisting and tongue playing after feeding, however, crib-biting was not recurrent. Why this behaviour did not show any correlations with colic occurrence remains unclear.

The occurrence of lameness but not colic was influenced by certain aspects of the horses’ personalities, and this influence was observed both with the questionnaire and behavioural data. Curious, playful and intelligent horses were more prone to develop lameness. The differences in the effects of personality traits on the occurrence of lameness found in the questionnaire and in the behavioural data showed that it is difficult to rely only on one type of personality assessment [82–83].

It is important to state that the results reflected the mean for the entire horse population and only from colic cases with unknown causes. In previous studies conducted within the same horse population, the number of colic cases was above the mean found in the literature [39–41]. Since the correlations between lameness and personality traits in the present study were low, results should be treated with caution and further studies with large sample sizes should be conducted.

## Conclusion

In this study, the most intelligent, curious and playful horses were the most susceptible horses to express abnormal behaviours and develop lameness. According to our results, passive, stubborn and confident horses were better able to cope with the demands of being a police horse. The classifying of horses according to their personalities could help in choosing the most suitable individuals for patrolling, thereby increasing animal welfare; however, this will need validating through long-term studies.

## Supporting information

S1 Fig Authorization letter from the Ethics Commission of Animals from Pontifícia Universidade Católica de Minas Gerais. (PDF)

S1 Table Dataset used for the analysis of the sham feeding test responses of the police horses. (XLSX)

S2 Table Dataset used for the analysis of the police horses’ personalities according to the questionnaires (sheet 1) and novel object (sheet 2), and the number of registers of colic and lameness in the police horses (sheet 3). (XLSX)
