# Supplementary material for: Personality, abnormal behaviour, and health: An evaluation of the welfare of police horses
Source: PLoS One. 2018 Sep 5;13(9):e0202750. doi: 10.1371/journal.pone.0202750 (PMC6124763; doi:10.1371/journal.pone.0202750)
Supplement: S1 Fig — (PDF) [file pone.0202750.s001.pdf]

PUC Minas
Pontifícia Universidade Católica de Minas Gerais
Pró-reitoria de Pesquisa e de Pós-graduação
Comissão de Ética no Uso de Animais - CEUA PUC Minas

20 May 2018.

The Ethics Commission of Animals use to research from Pontifícia Universidade Católica of Minas Gerais (PUC Minas) evaluated the project of Ivana Gabriela Schork, intitule “Behavioral Disorders and Personality: an evaluation of the welfare in horses”, concluding that this project did not need of recommendations from this Commission due its execution to be based only in observations of the routine of animals at police academy.

Gisele Pires M. Dantas

Gisele Pires de Mendonça Dantas

CEUA PUC Minas Coordinator

Av. Dom José Gaspar, 500 - Prédio 03, 2°. andar – sala 228 - Fone: 3319-4517
CEP 30535-610 - Belo Horizonte - Minas Gerais – Brasil
ceua@pucminas.br
